# Measurement of the mass-flow-rate characterization parameters of high-pressure pneumatic servo slide valves

**DOI:** 10.1038/s41598-022-07377-z

**Published:** 2022-02-28

**Authors:** Dijia Zhang, Longlong Gao, Shaoliang Zhou, Yuxuan Ma, Baoren Li

**Affiliations:** FESTO Pneumatics Centre, School of Mechanical Science and Engineering, Huazhong University of Science and Technology, Wuhan, 430074 China

**Keywords:** Mechanical engineering, Aerospace engineering

## Abstract

The mass-flow-rate characteristics of high-pressure pneumatic servo valves (HPSVs) have an important effect on the dynamic performance of high-pressure servo systems. However, these characteristics are difficult to obtain by theoretical calculations and flowmeter measurements owing to the compressibility of high-pressure gas. In this paper, a new measurement method of the mass-flow-rate characterization parameters of HPSVs is proposed based on the principle of the series connection sonic discharge of valve orifices. The effective cross-sectional area and critical pressure ratio of the servo valve orifices can be accurately and efficiently determined by connecting two valve orifices in series and exchanging the flow sequence of the two valve orifices. The two assumptions including the sonic and adiabatic discharge of the proposed measurement method were verified. A comparison between the test and simulation data showed that the accuracy of the measured effective cross-sectional area and critical pressure ratio of the HPSV was high. The measured critical pressure ratio ranged from 0.46 to 0.50, and the flow coefficient represented by the effective cross-sectional area variation decreased with increasing valve opening. These findings have general implications for the accurate design, analysis, and control of high-pressure pneumatic servo systems.

## Introduction

A high power ratio and instant expansibility of high-pressure gases can effectively improve the dynamic characteristics, increase the inherent frequency, and improve the response speed of pneumatic systems. At the same time, high pressure can enable miniaturization of the components, thus saving installation space and costs. Therefore, high pressure is applied in specialized fields, such as aviation, aerospace, military equipment, and drilling platforms^1–3^.

The performance of high-pressure pneumatic servo valves (HPSVs) is critical for system design, system control, and evaluation and optimization of the system performance^4,5^. Servo valves usually have a slide valve structure, and their flow characteristics are the basis for those of the entire high-pressure pneumatic servo system^6–9^. Many experts and researchers have been investigating the measurement of the flow characteristics of pneumatic components for many years, and have developed numerous flowmeters and measurement methods.

The flow characteristics of the servo valve are measured using different flowmeters depending on the magnitude of the flow rate. A precision hydraulic motor or vortex meter is used for testing at high flow rates, while a hydraulic cylinder is used for testing when the flow rate is not high^10^. In this method, the density and viscosity of the fluid have greatly affect the measurement results, and therefore, this method is more suitable for liquid media or low-pressure gases that exhibit little change in density. The ISO 6358 test method using sonic conductance and critical-pressure-ratio characterization of the mass flow rate can also achieve measurement of the flow characteristics^11^. However, this method has many provisions, stringent requirements for the accuracy of the test device and measurement instrumentation, and high test-gas consumption and cost^12,13^. Kuroshita and Oneyama^14^ proposed a hybrid method for measuring flow characteristics based on ISO 6358^11^ and JIS B 8390^15^ that can measure parts with large apertures using small devices. However, for components with relatively small critical pressure ratios, the errors in the measured sonic conductance are large, and this method cannot fully describe the mass flow rates of pneumatic components. Kawashima and co-workers^16,17^ proposed a method to measure the flow characteristics using an isothermal chamber. However, the method is influenced by the density of the filler, the isothermal chamber is difficult and expensive to fabricate, and the isothermal performance of the chamber is difficult to evaluate ^18,19^. Imamura et al. ^20^ proposed a gravimetric method called SRoGS that can be used to measure the gas mass flow rate within the range of 0.012–0.062 g/min in a vacuum chamber, but the measurement error is large at high gas flow rates, and this method requires a strict test environment. Kashan et al. ^21^ proposed a new mass-flow-rate sensing element that is particularly suitable for low-flow-rate measurement in vacuum processing systems. These measurement methods suffer from high measurement device requirements and difficulty in measurement, and they are only applicable to low-pressure pneumatic components. High-pressure pneumatic measurements require a simple and efficient measurement method owing to the high differential pressure, high variation in the gas density, and high flow rate.

The Chinese national standard GB/T 14513 developed the series connection sonic discharge method ^22^. In this method, the mass-flow-rate characteristics of the component can be obtained indirectly by measuring the effective cross-sectional area and critical pressure ratio in the choked flow state by using only the specified cavity with two identical components connected in series in the test system. For components with a critical pressure ratio greater than 0.25, the effective cross-sectional area and critical pressure ratio obtained from the measurement are more credible, while for small critical pressure ratios and low inlet pressures, the measurement results will be more inaccurate ^23^. Therefore, this method is suitable for high-pressure pneumatics and has the advantages of low test-gas consumption, low cost, and high efficiency ^24^. Gao et al. ^25^ connected two high-pressure solenoid valves in series and obtained high-accuracy effective cross-sectional area and critical pressure ratio values by the series connection sonic discharge method when the discharge time was less than 2 s.

According to the slide valve structure characteristics of the HPSV investigated in this study, the two control orifices of the servo valve are equivalent to the throttle orifices, which can be used to measure the mass flow rate by using the principle of the series connection sonic discharge method. Based on the principle of mass conservation of the gas flowing through the two tandem throttle orifices and making two assumptions, that is, that the discharge process is adiabatic and that the flow rate at the downstream throttle orifices is sonic, a new method to accurately and efficiently measure the effective cross-sectional area and critical pressure ratio of the servo valve orifices is derived from principle. This new method requires only exchanging the flow sequence of two valve orifices and then separating the measurement of the gas pressure change and temperature in the chamber. In this way, the mass-flow-rate characteristics of the HPSV can be indirectly measured. Regarding the assumptions of the method, computational fluid dynamics (CFD) is used to obtain the internal flow field of the HPSV whose valve orifice is connected in series to verify the assumption that the downstream throttle flow rate is the sonic speed. The simulation with no heat exchange and the test results during the discharge process are compared to verify that the adiabatic assumption holds.

## Principle of measurement of the mass-flow-rate characterization parameters

### Throttle orifice gas mass flow rate equation

According to the international standard ISO 6358^11^ on the mass-flow-rate equation equivalent to an elliptic equation, the mass flow rate of gas flowing through each throttle orifice can be expressed as

1 $$Q_{m\max } = Sp_{1} \sqrt {\frac{kR}{{T_{1} }}} \sigma_{cr}^{{\frac{k + 1}{{2k}}}} = 0.0404S\frac{{p_{1} }}{{\sqrt[{}]{{T_{1} }}}},0 \le \frac{{p_{2} }}{{p_{1} }} \le \sigma_{cr}$$

2 $$Q_{m} = Q_{m\max } \sqrt {1 - \left[ {\frac{{(p_{2} /p_{1} ) - \sigma_{cr} }}{{1 - \sigma_{cr} }}} \right]^{2} } ,\sigma_{cr} \le \frac{{p_{2} }}{{p_{1} }} \le 1$$

where *Q*_*m*_ is the maximum mass flow rate in the choked-flow state, *S* is the effective cross-sectional area of the rectangular valve orifices, *σ*_*cr*_ is the critical pressure ratio in the choked-flow state, *p*_*1*_ is the upstream pressure, *p*_*2*_ is the downstream pressure, *T*_*1*_ is the upstream temperature, *R* is the gas constant (*R*=287 J/(kg∙K)), and *k* is the adiabatic index (*k*=1.4). From Eqs. (1) and (2), the mass-flow-rate characteristics of the HPSV can be fully expressed by using two characteristic parameters: the effective cross-sectional area *S* and the critical pressure ratio in the choked-flow state *σ*_*cr*_.

### HPSV structure and measurement method

**Figure 1:**
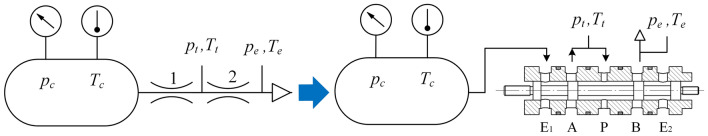
Equivalent schematic of the HPSV series connection sonic discharge method.

The HPSV investigated in this study was a three-position five-way valve, which contains five valve orifices, two of which are the main control orifices (valve orifices A and B). Based on an analogy to four-sided slide valves, the servo valve orifices are equivalent to the throttle orifices. After passing through valve orifices A and B, the high-pressure gas can then be discharged into the atmosphere from the chamber according to the series connection sonic discharge method. The equivalent schematic of the series connection sonic discharge method is shown in Fig. 1.

### Assumptions

 (a) Sonic assumption. Valve orifices A and B are connected in series, and the through-flow areas are the same or very similar to ensure that the high-pressure gas is subsonic in valve orifice A and sonic in valve orifice B; that is, the critical section is at valve orifice B.

(b) Adiabatic assumption. Under the condition that the frictional heat exchange between the gas and the tube wall is ignored and the discharge time is as short as possible, the temperature inside the chamber is maintained; it is the same as that of the outside environment (i.e., *Tc* and *Tt*) during the discharge process.

### Effective cross-sectional area *S*

The effective cross-sectional area of high-pressure pneumatic components *S* is measured by the series connection sonic discharge method. *S* is calculated by considering the thermodynamic equations of the adiabatic-discharge process and isovolumetric process, and the gas dynamics equation:

3 $$S = 26.1\frac{V}{t}\sqrt {\frac{273}{{T_{0} }}} \left[ {\left( {\frac{{p_{10} }}{{p_{\infty } }}} \right)^{\frac{1}{5}} - 1} \right]$$

### Critical pressure ratio *σ*_*cr*_

The mass flow rate through the valve orifices is

4 $${\text{Q}}_{{{\text{m}}AB}} = 0.0404{\text{S}}_{AB} \frac{{{\text{p}}_{{\text{c}}} }}{{\sqrt {{\text{T}}_{{\text{c}}} } }}$$

5 $${\text{Q}}_{{{\text{mB}}}} = 0.0404{\text{S}}_{B} \frac{{{\text{p}}_{{\text{t}}} }}{{\sqrt {{\text{T}}_{{\text{t}}} } }}$$

where *Q*_*m*AB_ is the mass flow rate in the case of throttle orifices A and B in the series connection, *Q*_*m*B_ is the mass flow rate of throttle orifice B, *S*_*AB*_ is the effective cross-sectional area in the case of throttle orifices A and B in the series connection, and *S*_*B*_ is the effective cross-sectional area of throttle orifice B. From the continuity equation, *Q*_*m*AB_ = *Q*_*m*B_ is obtained, and according to the adiabatic assumption, *T*_*c*_ = *T*_*t*_ is obtained. Thus,

6 $$\frac{{{\text{p}}_{{\text{t}}} }}{{{\text{p}}_{{\text{c}}} }} = \frac{{{\text{S}}_{AB} }}{{{\text{S}}_{B} }}$$

From the international standard ISO6358^11^,

7 $$Q_{{mA}} = 0.0404\frac{{P_{c} }}{{\sqrt {T_{c} } }}\sqrt {1 - \left( {\frac{{p_{c} /p_{t} - \sigma _{{Acr}} }}{{1 - \sigma _{{Acr}} }}} \right)^{2}}$$

Similarly, the continuity equation gives *Q*_*mA*_ = *Q*_*mB*_, so

8 $$\frac{{p_{t} }}{{p_{c} }} = \frac{{S_{A} }}{{S_{B} }}\sqrt {1 - \left( {\frac{{p_{c} /p_{t} - \sigma_{Acr} }}{{1 - \sigma_{Acr} }}} \right)^{2} }$$

Thus, from the above equation,

9 $$\frac{{{\text{S}}_{{AB}} }}{{{\text{S}}_{B} }} = \frac{{\sigma _{{Acr}} + 1 - \sigma _{{Acr}} \sqrt {1 + 1 - 2\sigma _{{Acr}} S_{B} /S_{A} ^{2} } }}{{1 + 1 - \sigma _{{Acr}} ^{2} S_{B} /S_{A} ^{2} }}$$

Equation (9) shows that the effective cross-sectional area of the series gas circuit is related only to the characterization parameters of the series-connected components, and it is independent of the pressure ratio between their two ends. The above equation then gives

10 $$\frac{{S_{AB} }}{{S_{A} }} = \sqrt {1 - \left( {\frac{{p_{t} /p_{c} - \sigma_{Acr} }}{{1 - \sigma_{Acr} }}} \right)^{2} }$$

Substituting Eq. (6) into Eq. (10) gives

11 $$\frac{{S_{AB} }}{{S_{A} }} = \sqrt {1 - \left( {\frac{{S_{AB} /S_{B} - \sigma_{Acr} }}{{1 - \sigma_{Acr} }}} \right)^{2} }$$

The critical pressure ratio of throttle orifice A is then

12 $${\upsigma }_{A{\rm cr}} = \frac{{{\text{S}}_{AB} /{\text{S}}_{B} - \sqrt {1 - \left( {{\text{S}}_{AB} /{\text{S}}_{A} } \right)^{2} } }}{{1 - \sqrt {1 - \left( {{\text{S}}_{AB} /{\text{S}}_{A} } \right)^{2} } }}$$

Exchanging the positions of throttle orifices A and B, the same critical pressure ratio of throttle orifice B can be obtained:

13 $${\upsigma }_{{{\text{Bcr}}}} = \frac{{{\text{S}}_{BA} /{\text{S}}_{A} - \sqrt {1 - \left( {{\text{S}}_{BA} /{\text{S}}_{B} } \right)^{2} } }}{{1 - \sqrt {1 - \left( {{\text{S}}_{BA} /{\text{S}}_{B} } \right)^{2} } }}$$

Based on the sonic discharge method, the pressure change and steady-state temperature of the gas in the constant-volume cavity are measured separately, and the effective cross-sectional area of each valve orifice *S* can be derived from Eq. (3) by considering the thermodynamic equations of the adiabatic-discharge process and isovolumetric process and the gas kinetics equation. From Eqs. (12) and (13), two characterization parameters, the effective cross-sectional area *S* and critical pressure ratio *σ*_*cr*_, can completely express the mass-flow-rate characteristics of the HPSV.

### Test measurements

**Figure 2:**
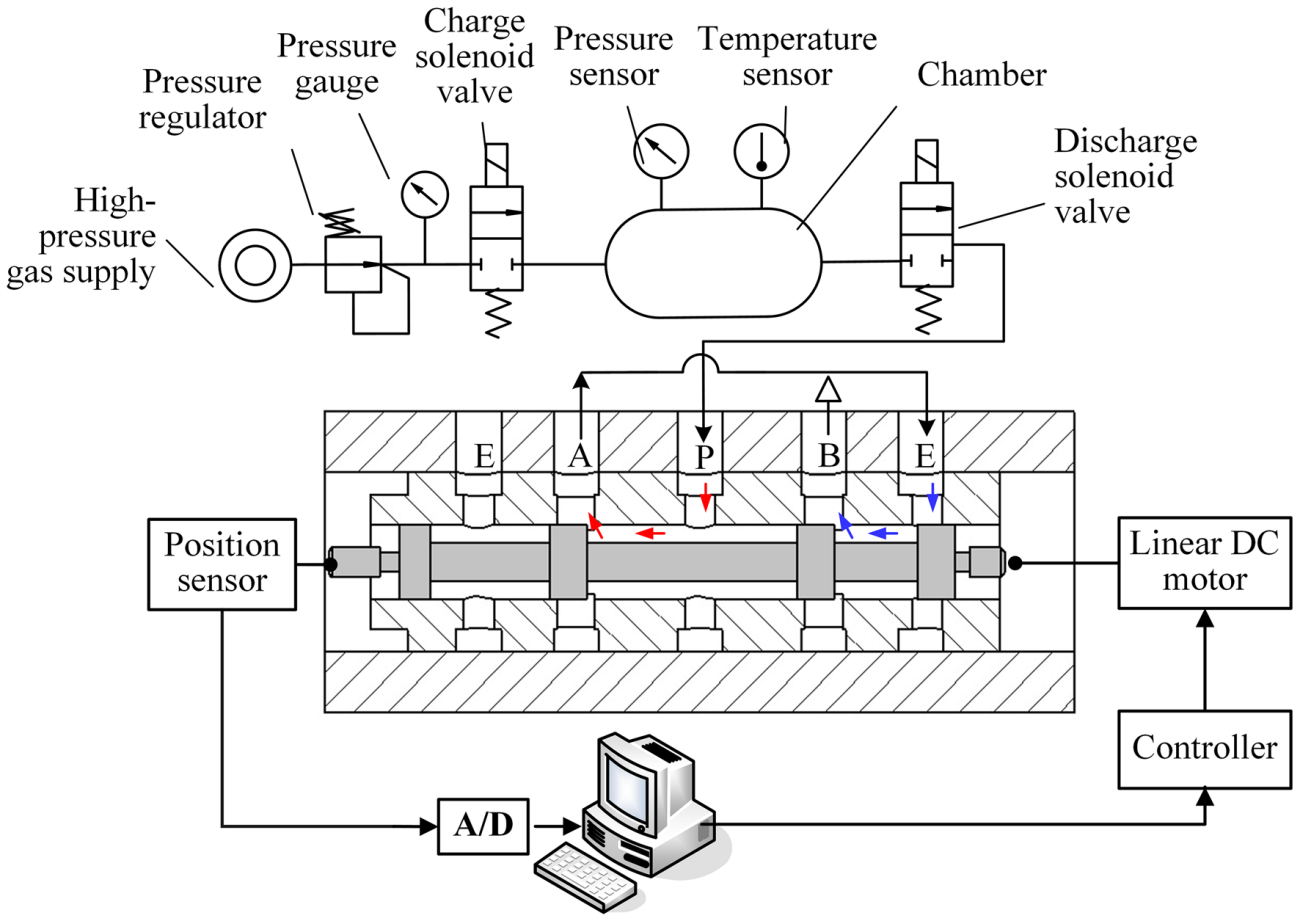
Schematic of the measurement of mass-flow-rate characterization parameters of the HPSV.

**Figure 3:**
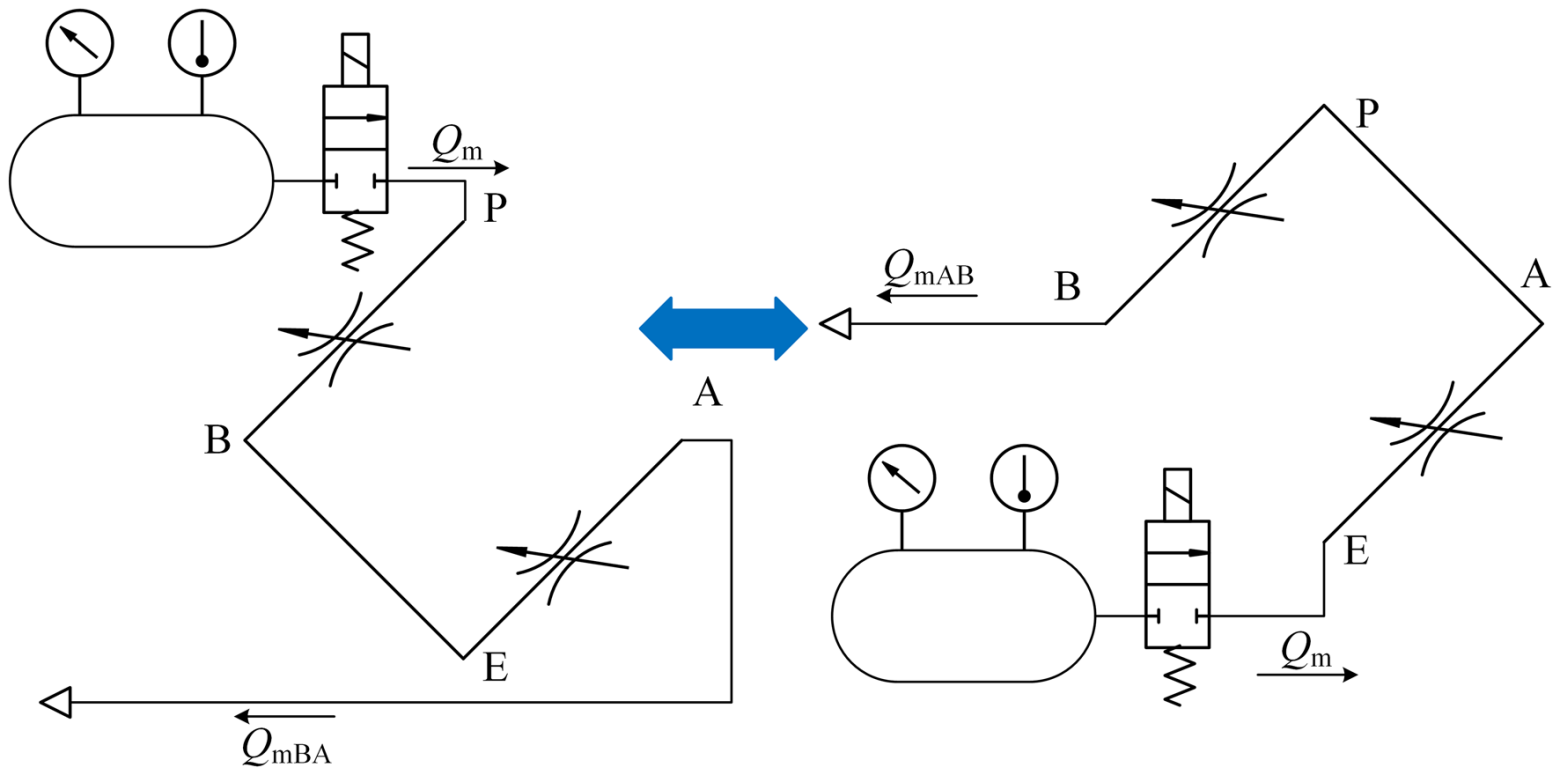
Schematic of the valve orifice series connection.

Based on the method, a test rig was designed, the schematic diagram of which is shown in Fig. 2. The chamber pressure sensor was used to measure the pressure of the constant-volume chamber, the chamber temperature was room temperature, the different valve orifices of the HPSV were connected with the chamber by the solenoid switch valve, the displacement sensor was used to detect the spool displacement of the HPSV, and the data were collected with an industrial control computer. For measurement, the valve orifices of the HPSV have two types of series connections, as shown in Fig. 3.

**Figure 4:**
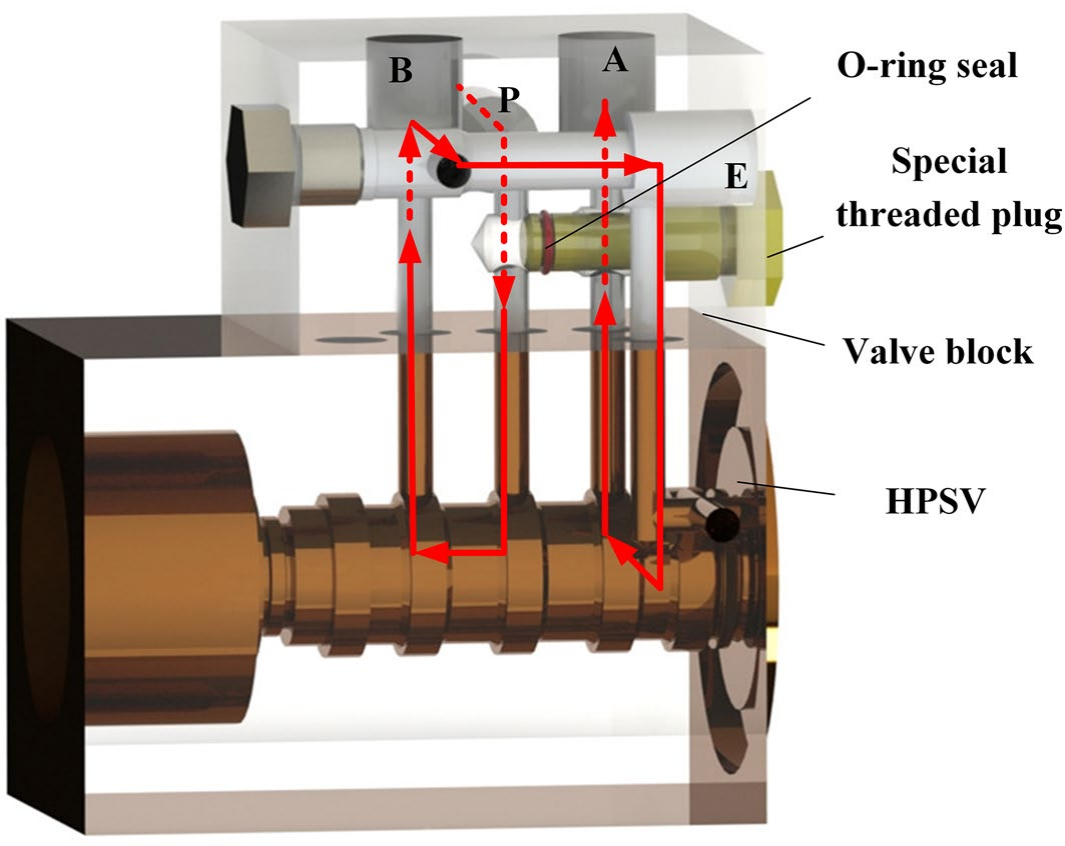
3D model of the valve block.

A valve block was designed to connect two valve orifices in series. To reduce manufacturing costs, a one-piece design was used, and a special threaded plug was designed to accompany this design. For a specific connection, the other lines were closed by O-ring seals. One of the tandem bridge connection configurations is shown in Fig. 4.

**Figure 5:**
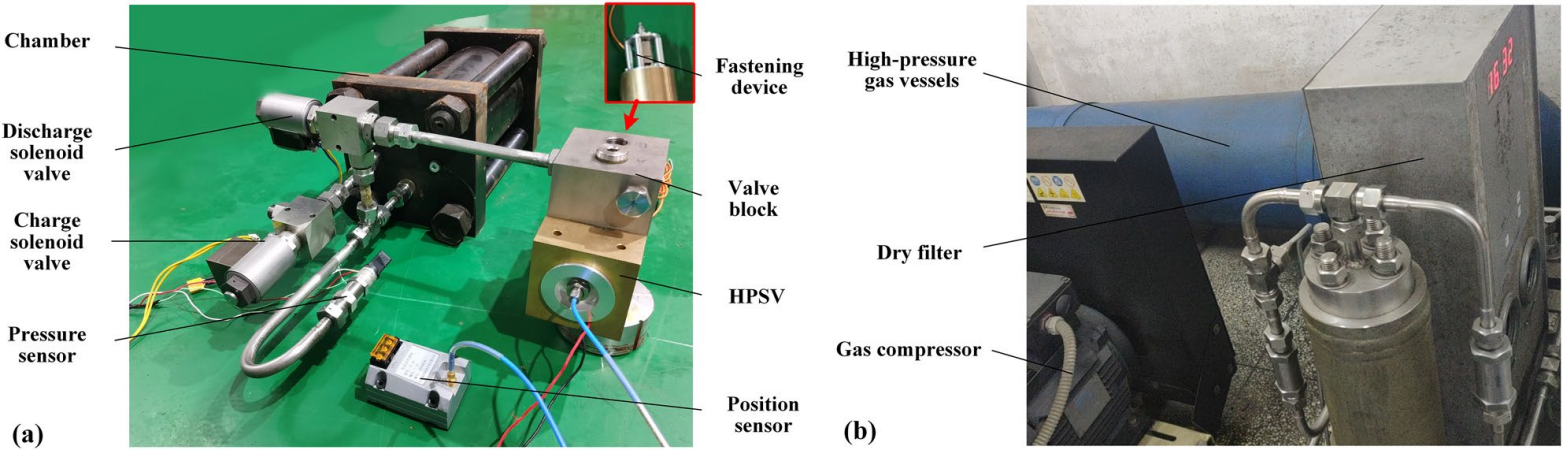
Photograph of the test components for the HPSV mass-flow-rate characterization parameters: **(a)** the chamber and test components, **(b)** high-pressure gas-supplying device.

The test rig was built (shown in Fig. 5) according to the schematic diagram of the measurement system, with a high-pressure gas cylinder used as the gas source and an industrial control computer used to control the solenoid valves and for data acquisition. A spool position fastening device was designed to help stabilize the spool displacement in the test by means of a positioning screw. The fastening device was mounted on the valve block and connected to the spool by threads. The list of related components is shown in Table 1.

**Table 1:** Parameters of the test components.

Name	Main parameters	Name	Main parameters
HPSV	Nominal diameter: 6 mm Maximum working pressure: 25 MPa	Pressure sensor	Range: 0–21 MPa Accuracy: 0.1%
PCL-816	Resolution: 16 bits Accuracy: 0.003% FS ± 1 LSB	Position sensor	Range: 0–3 mm Accuracy: 0.1%
Chamber	Maximum working pressure: 24 MPa Volume: 1.6 L	Solenoid valve	Nominal diameter: 8 mm Response time: < 150 ms Maximum working pressure: 18 MPa

**Figure 6:**
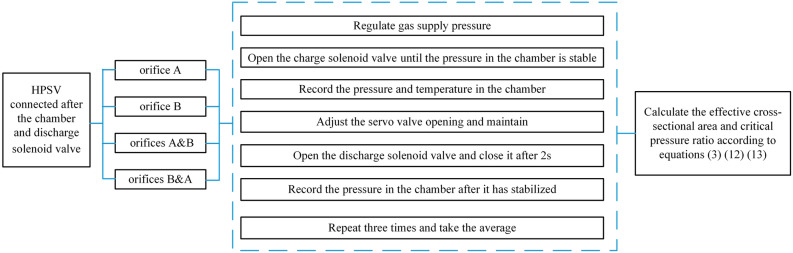
Test measurement procedure.

According to the abovementioned method, the internal pressure values of the pressure chamber after discharging were measured for four connection cases: valve orifice A, valve orifice B, tandem valve orifices A and B, and tandem valve orifices B and A. The measurement procedure is shown in Fig. 6.

**Figure 7:**
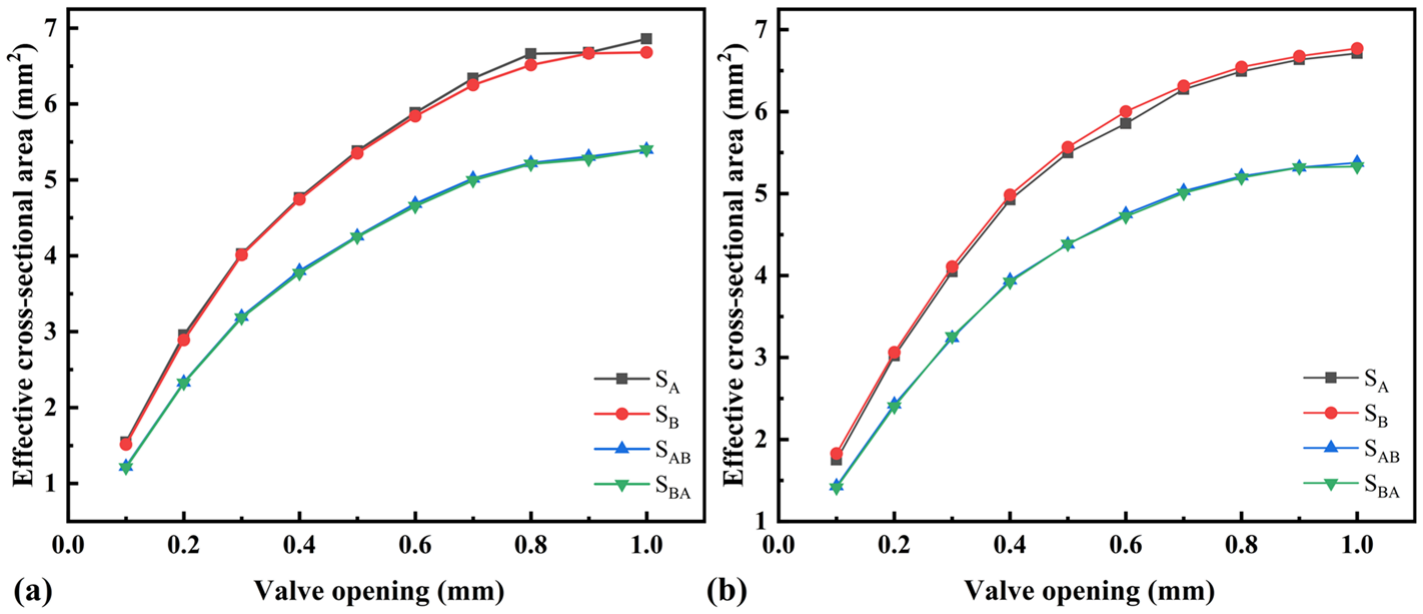
Effective cross-sectional area: initial pressure of the chamber is **(a)** 5 MPa; **(b)** 15 MPa.

**Figure 8:**
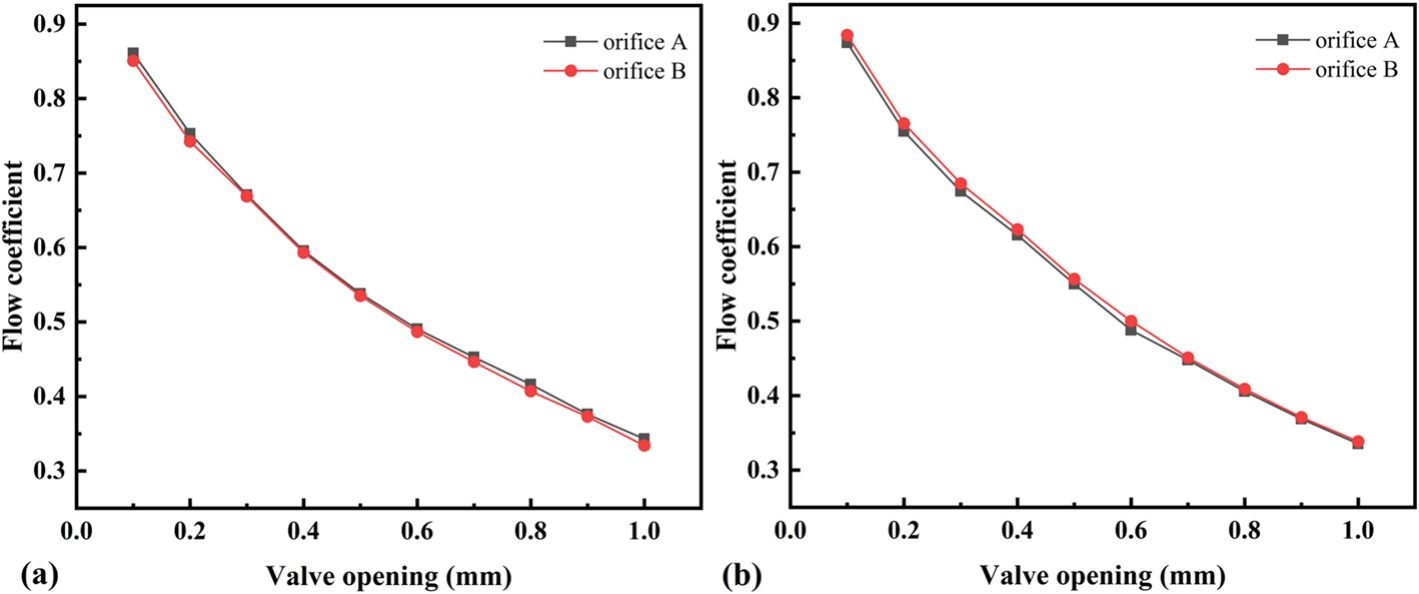
Flow coefficient: initial pressure of the chamber is **(a)** 5 MPa; **(b)** 15 MPa.

A discharge time of 2 s can be considered an adiabatic process^25^. The test was conducted at initial chamber pressures of 5 and 15 MPa, and the mass-flow-rate measurements were repeated several times for valve openings of 0.1–1 mm. The effective cross-sectional areas of each valve opening under the two pressure conditions were calculated according to Eq. (3), where *S*_*A*_, *S*_*B*_, *S*_*AB*_, and *S*_*BA*_ are the effective cross-sectional areas of valve orifice A, valve orifice B, tandem valve orifices A and B, and tandem valve orifices B and A, respectively, as shown in Fig. 7. The flow coefficient *C*_*d*_ is the ratio of the effective cross-sectional area to the geometric cross-sectional area, and it is an important parameter for evaluating the flow capacity of control valve orifices:

14 $$C_{d} = \frac{S}{{4bx_{v} }}$$

where *b* is the side length of the rectangular orifice (*b* = 5 mm in this study) and *x*_*v*_ is the opening of the valve orifice. The flow coefficients of valve orifices A and B measured under the two pressure conditions are shown in Fig. 8. Based on the value and the law of the flow coefficient, the measurement of the effective cross-sectional area is more accurate.

**Figure 9:**
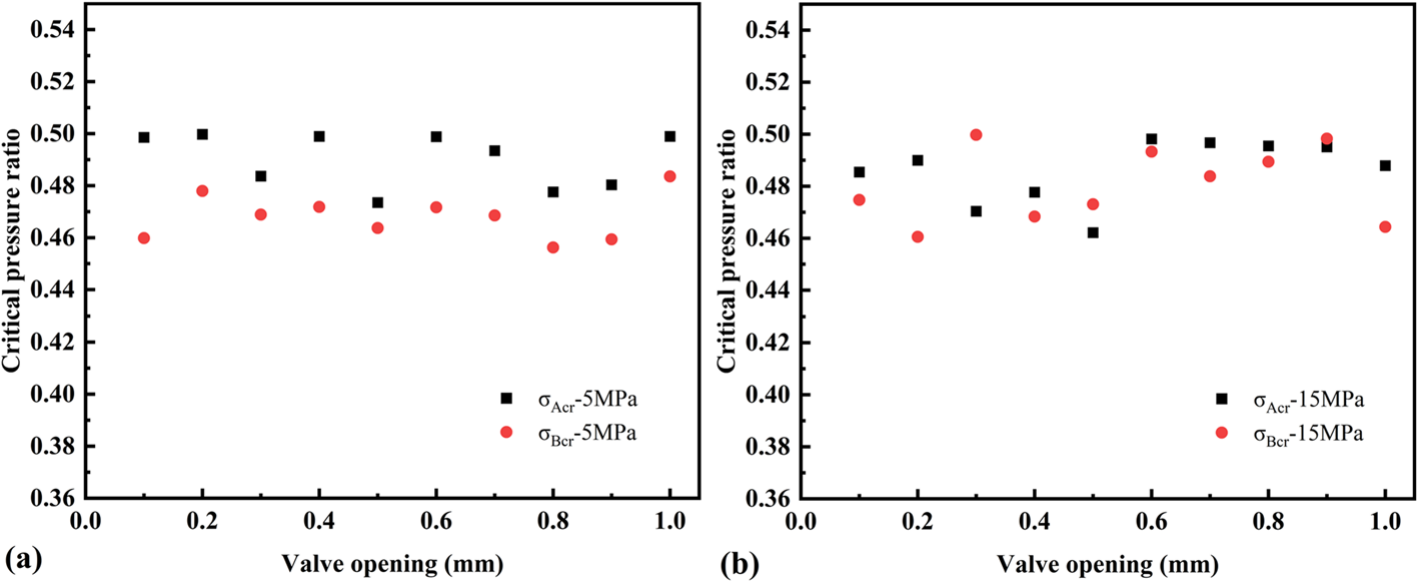
Critical pressure ratio: initial pressure of the chamber is **(a)** 5 MPa; **(b)** 15 MPa.

**Figure 10:**
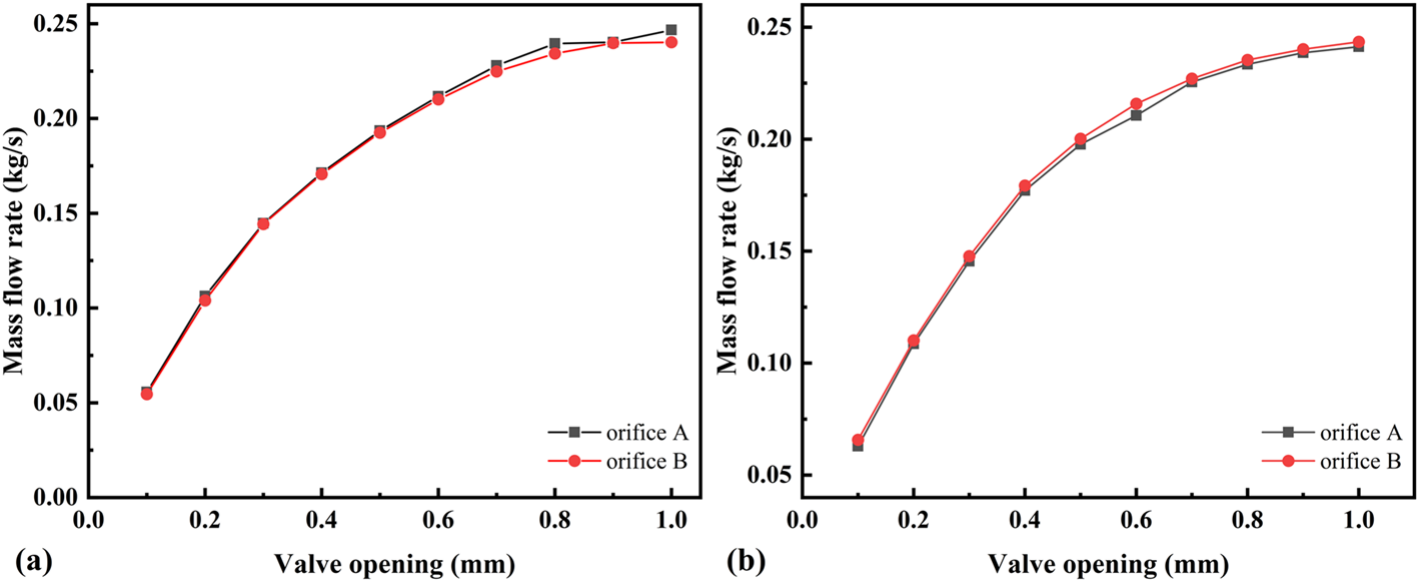
Mass-flow-rate characteristics: initial pressure of the chamber is **(a)** 5 MPa; **(b)** 15 MPa.

According to calculations with Eqs. (12) and (13), the critical pressure ratios of valve orifices A and B were obtained under two pressure conditions (Fig. 9). The test critical pressure ratio was between 0.46 and 0.50, which is in line with the critical pressure ratio rule for general pneumatic components. Under the two pressure conditions, the *P*_*2*_*/P*_*1*_ values were both less than the test critical pressure ratio. Therefore, the measured effective cross-sectional areas *S*_*A*_ and *S*_*B*_ were substituted into Eq. (1) to obtain the mass flow rate of the two valve orifices (Fig. 10).

### Assumption verification

#### Verification of the sonic assumption

**Figure 11:**
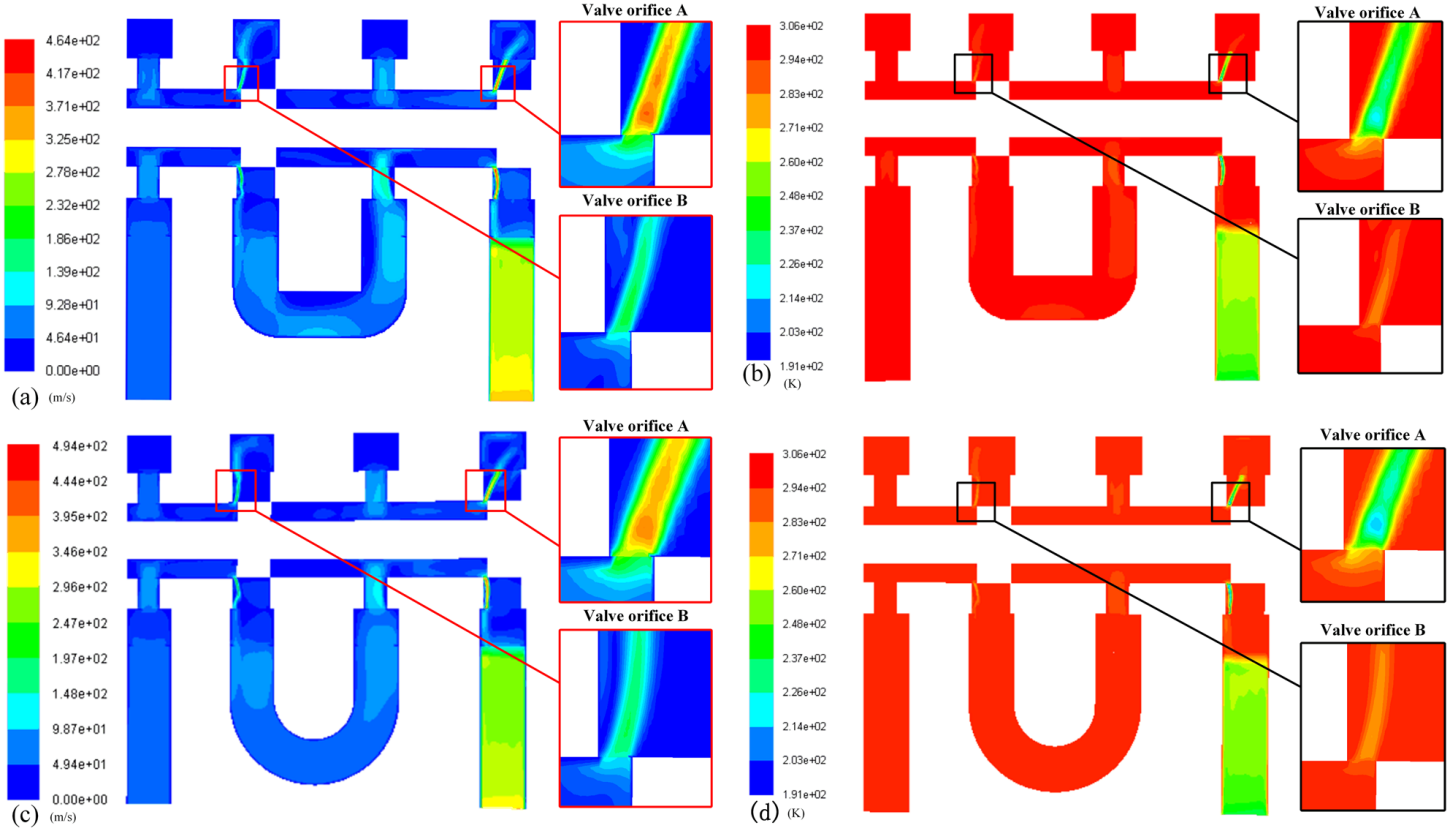
Gas flow contour of the tandem valve orifices: inlet pressure is 5 MPa, **(a)** velocity contour, **(b)** temperature contour; inlet pressure is 15 MPa, **(c)** velocity contour, **(d)** temperature contour.

For the assumption that the flow velocity at the downstream valve orifice is the speed of sound in the mass-flow-rate characterization parameters measurement method of HPSVs, the CFD method was used to study the gas flow through the two valve orifices^26^. CFD is known as a robust method for predicting fluid dynamics parameters and the most common software is ANSYS/fluent ^27,28^. As shown in Fig. 11, the geometric areas of valve orifices A and B in the model were the same. The results showed that under the two pressure conditions, the gas flow at valve orifice A was subsonic (the highest velocities were 186 and 197 m/s) and that at valve orifice B was sonic (flow rates of 325 and 323 m/s). Here, it should be noted that the high-velocity jet at the valve orifice causes a drop in temperature, so the local sonic speed is reduced. According to the temperatures (263 and 260 K) at valve orifice B, the sonic flow rates at these temperatures were 325 and 323 m/s, respectively; that is, the flow velocity at valve orifice B reached sonic speed. After passing through valve orifice B, the flow rate reached supersonic speed because of the expanded through-flow area, similar to the Rafael nozzle structure. Therefore, the sonic assumption is valid.

#### Verification of the adiabatic assumption

To verify the accuracy of the mass-flow-rate test results of the HPSV and the assumption of adiabatic heat inside the chamber during the discharge process, the pressure change inside the chamber during 2 s discharge was investigated by MATLAB/Simulink. The pressure and temperature variation of high-pressure gas inside the chamber can be written as follows,

15 $$\frac{dp}{{dt}} = \frac{RT}{V}\frac{dm}{{dt}} + \frac{p}{T}\frac{dT}{{dt}} = - \frac{RT}{V}Q_{m} + \frac{p}{T}\frac{dT}{{dt}}$$

16 $$\frac{dT}{{dt}} = \frac{{h_{o} \cdot A\left( {T_{a} - T} \right)}}{{C_{v} \cdot m}} - \frac{{\left( {k - 1} \right)T}}{m}Q_{m}$$

where *A* is the heat transfer area, *h*_*0*_ is the heat transfer coefficient, *T*_*a*_ is the chamber wall temperature, *T* is the gas temperature in the chamber, *C*_*V*_ is the specific heat at constant volume, and *m* is the gas mass. From Eqs. (15) and (16), it can be seen that during the discharge process, the variation in the pressure and temperature of the high-pressure gas are affected by the mass flow rate *Q*_*m*_ and the heat transfer coefficient *h*_*0*_, and if this process is very short, it can be considered an adiabatic process. According to this assumption, the heat-transfer coefficient inside the chamber during the discharge process was 0. In performing the simulation, the test critical pressure ratio and effective cross-sectional area were substituted for the theoretical values. The test data of the pressure change inside the chamber during the discharge process were compared with the simulated data; if the results are very close, the heat transfer coefficient can be considered to be 0, which means that the adiabatic assumption is valid.

**Figure 12:**
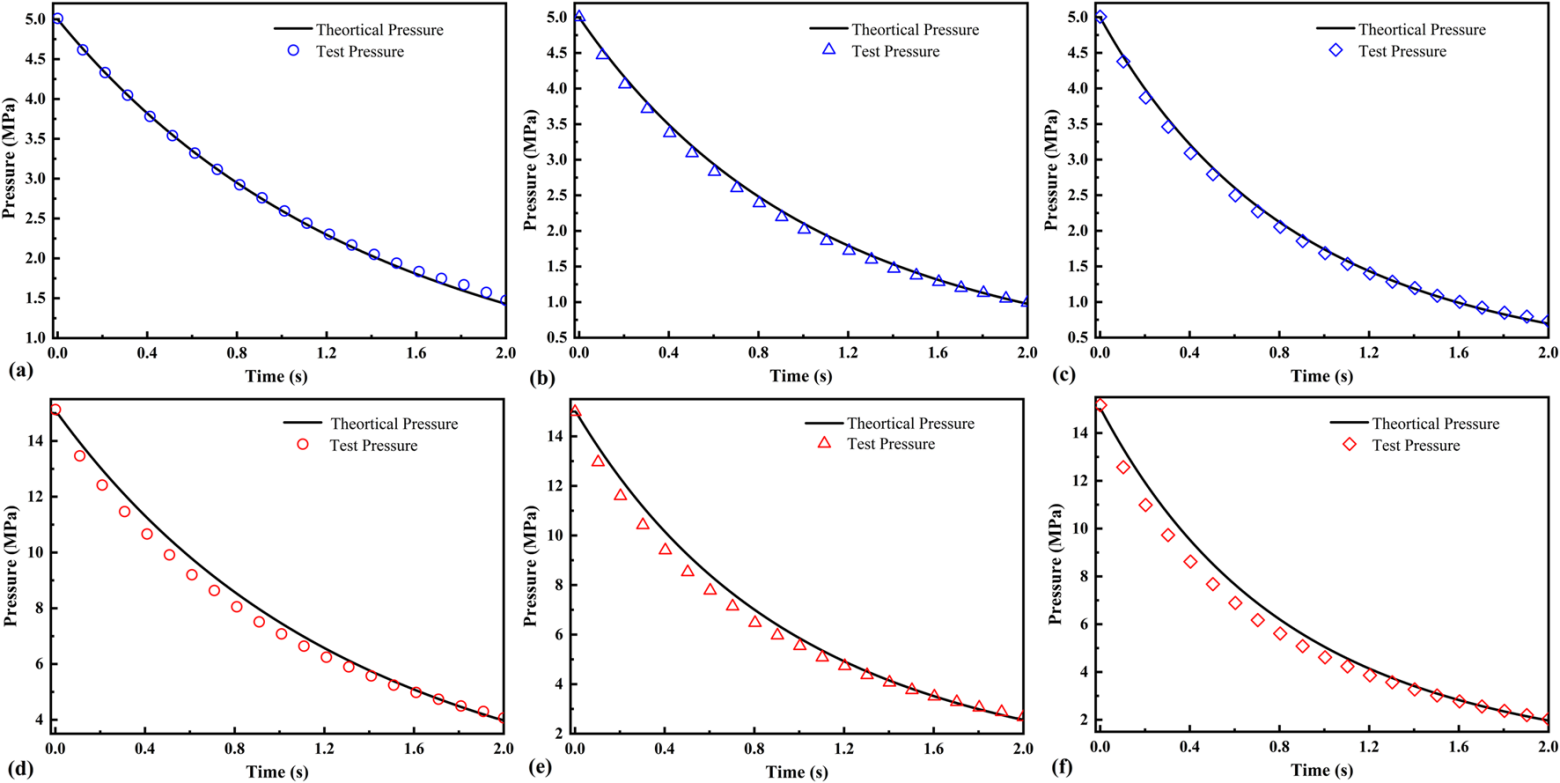
Pressure in the constant-volume chamber during discharge: initial pressure of the chamber is 5 MPa, the valve opening is **(a)** 0.3 mm, **(b)** 0.5 mm, **(c)** 0.8 mm; initial pressure of the chamber is 15 MPa, the valve opening is **(d)** 0.3 mm, **(e)** 0.5 mm, **(f)** 0.8 mm.

Comparing the pressure changes for valve openings of 0.3, 0.5, and 0.8 mm, the results showed that the simulated data were in good agreement with the test data when the discharge time was 2 s under an initial pressure of 5 MPa (Fig. 12). The test data values were slightly smaller than those of the simulated data under an initial pressure of 15 MPa for less than 1.6 s, and higher after 1.6 s. The heat transfer between the high-pressure gas inside the chamber and the chamber wall during discharge can be expressed based on the mixing theory of natural and forced convection ^29–31^. When the pressure inside the chamber increases, the heat-transfer coefficient accordingly increases, and the convective heat-transfer intensity in the chamber increases. Therefore, the discharge time can be slightly reduced to ensure that the adiabatic assumption holds.

**Figure 13:**
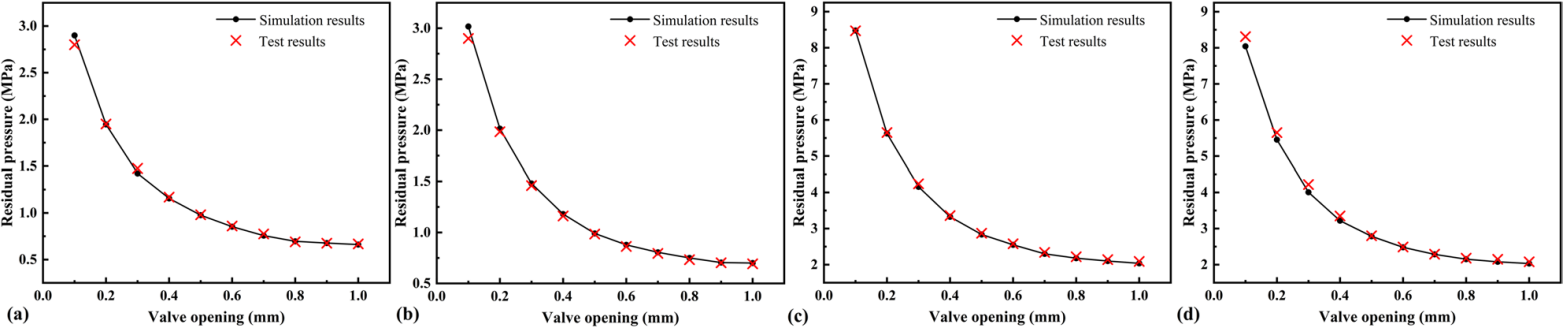
Residual pressure in the constant-volume chamber after discharge: initial pressure 5 MPa **(a)** valve orifice A; **(b)** valve orifice B; initial pressure 15 MPa; **(c)** valve orifice A; **(d)** valve orifice B.

The simulation and test results of the residual pressure after discharge for 2 s are compared in Fig. 13. The simulation results were in good agreement with the test results, and the maximum relative error was 0.05. Therefore, the adiabatic assumption holds, and the effective cross-sectional area and critical pressure ratio have high accuracy.

## Discussion

**Figure 14:**
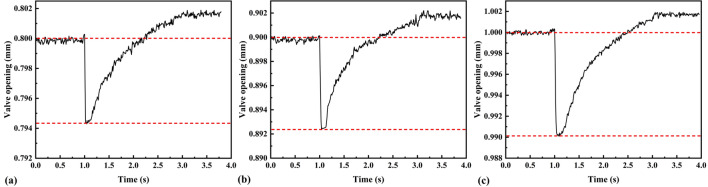
Spool displacement monitoring for different valve openings: **(a)** 0.8 mm; **(b)** 0.9 mm; **(c)** 1.0 mm. Discharge starts at 1 s.

This paper presents a test method to determine the mass-flow-rate characterization parameters of a slide-valve-type HPSV. The test results are shown in Figs. 7, 8, 9 and 10. The relationship between the valve opening and mass flow rate (Fig. 10) was that the mass flow rate increased at a decreasing rate as the valve opening increased. In particular, the mass-flow-rate increase significantly decreased when the valve opening was greater than 0.5 mm. The reason is that the flow coefficient is smaller and the gas flow force is larger for a larger orifice opening ^32^. When the mass flow rate at the valve orifices increases to the power domain limit at the moment of discharge, the gas flow force acting on the spool increases to a level comparable with the electromagnetic force of the motor. This phenomenon makes the spool appear uncontrollable for a short time, and the gas flow force will make the valve orifices smaller, resulting in a reduced flow rate through the orifices. Although a fastening device was added to help the spool compensate for some of the gas flow force, the gas flow force still has a large effect on the spool displacement when the valve opening is large, as shown in Fig. 14.

As a servo valve is a high-precision control element, its mass-flow-rate characteristics are very important. The measured mass-flow-rate characteristics help to further optimize those nonlinearity by improved control algorithms. It was found that the gas flow force significantly affects the spool position, which can be reduced by further optimizing the control algorithm to improve the servo valve's resistance to gas flow force. In addition, in the high-pressure pneumatic servo system, the influence of the nonlinearity of the mass flow rate on the system control performance can be analysed, and the control compensation method can be studied.

## Conclusion

The new measurement method proposed in this paper is based on the series connection sonic discharge method, where two valve orifices are equated to two throttle orifices. The effective cross-sectional area and the critical pressure ratio can be obtained by exchanging the flow sequence of the two valve orifices, and these two parameters are used to characterize the mass flow rate of a slide-valve-type HPSV. A three-position five-way HPSV is used for the test.

According to the test data under two pressure conditions, the flow coefficient decreased with increasing valve orifice opening, and the flow coefficients were very close for the same valve opening. The critical pressure ratio ranged from 0.46–0.50, which is in accordance with the critical pressure ratio rule of general pneumatic components. The measured mass flow rate and valve opening showed a nonlinear relationship owing to the different orifice resistance values at different openings and the influence of the instantaneous aerodynamic force of the discharge.

The measurement method has two assumptions: the sonic assumption and the adiabatic assumption. The sonic assumption was verified by CFD, and the simulation data were compared with the test results to confirm that the adiabatic assumption holds for the 2 s discharge. It was found that under high-pressure conditions, the discharge time needs to be reduced accordingly to ensure the adiabaticity of the discharge process, and it is recommended to reduce the discharge time to 1.6 s in the high-pressure measurement.

The proposed method for testing the mass-flow-rate characterization parameters of high-pressure pneumatic servo slide valves is effective and feasible. This method has the advantages of a simple structure, low test-gas cost, high efficiency, and energy savings. This measurement method is also applicable to other slide-valve-type high-pressure pneumatic valves. It is recommended to consider the influence of the gas flow forces during the measurement, and more accurate measurements can be obtained by increasing the valve’s resistance to interference.

## References

[1] Nie S, Liu X, Yin F, Ji H, Zhang J (2018). Development of a high-pressure pneumatic on/off valve with high transient performances direct-driven by voice coil motor. Appl. Sci.-Basel..

[2] Dong D, Li X (2015). Development of a novel parallel-spool pilot operated high-pressure solenoid valve with high flow rate and high speed. Chinese J. Mech. Eng..

[3] Liu G, Wang J, Dang K, Yuan S (2015). Effects of flow stress behaviour, pressure loading path and temperature variation on high-pressure pneumatic forming of ti-3al-2.5v tubes. Int. J. Adv. Manuf. Technol..

[4] Yan H, Liu Y, Ma L (2019). Mechanism of temperature’s acting on electro-hydraulic servo valve. IEEE Access..

[5] Zhang S, Li S (2015). Cavity shedding dynamics in a flapper–nozzle pilot stage of an electro-hydraulic servo-valve: Experiments and numerical study. Energy Conv. Manag..

[6] Briones J, Espulgar W, Koyama S (2021). A design and optimization of a high throughput valve based microfluidic device for single cell compartmentalization and analysis. Sci. Rep..

[7] Heinken H, Ulrich S, Bruns R, Schneider S (2019). High-response electrorheological servo valve. J. Intell. Mater. Syst. Struct..

[8] Pawananont K, Leephakpreeda T (2018). Sequential control of multichannel on–off valves for linear flow characteristics via averaging pulse width modulation without flow meter: An application for pneumatic valves. J. Dyn. Syst. Meas. Control-Trans. ASME..

[9] Tan C, Wu H, Dong F (2013). Mass flow rate measurement of oil-water two-phase flow by a long-waist cone meter. IEEE Trans. Instrum. Meas..

[10] ISO 10770-2:2012. *Hydraulic Fluid Power—Electrically Modulated Hydraulic Control Valves—Part 2: Test Methods for Three-Port Directional Flow-Control Valves*. (2012)

[11] ISO 6358-1-2013. *Pneumatic Fluid Power—Determination of Flow-Rate Characteristics of Components Using Compressible Fluids—Part 1: General Rules and Test Methods for Steady-State Flow*. (2013)

[12] Y. Fan, L. Gangyan, H. Jian, L. Wengfeng, Q. Wang. Method for resultant and calculating the flow-rate characteristics of pneumatic circuit. in *2015 International Conference on Fluid Power and Mechatronics (FPM)*. 379–384. 10.1109/FPM.2015.7337144. (IEEE, 2015).

[13] Righettini P, Giberti H, Strada R (2013). A novel in field method for determining the flow rate characteristics of pneumatic servo axes. J. Dyn. Syst. Meas. Control-Trans. ASME..

[14] Kuroshita, K. & Oneyama, N. Improvements of test method of flow-rate characteristics of pneumatic components. in *SICE 2004 Annual Conference*. 147–152. https://ieeexplore.ieee.org/document/5332874. (IEEE, 2004).

[15] JIS B8390-2000. *Pneumatic Fluid Power Components Using Compressible Fluids Determination of Flow Rate Characteristics*. (2000)

[16] Kawashima K, Ishii Y, Funaki T, Kagawa T (2004). Determination of flow rate characteristics of pneumatic solenoid valves using an isothermal chamber. J. Fluids Eng. Trans. ASME..

[17] T. Funaki, T. Kato, K. Kawashima, T. Kagawa. Study on a performance evaluation of oscillatory gas flow generator with highly precise inlet flow control system. in *SICE 2009 Annual Conference*. 3819–3824. https://ieeexplore.ieee.org/document/5332874. (IEEE, 2009)

[18] Yang L, Gan Y, Liu P (2013). Study on heat transfer of porous media for isothermal chamber. Exp. Therm. Fluid Sci..

[19] Yang L, Shen H, Ye Q, Liu C (2017). Influence of the distribution of porous media on the heat conduction enhancement and isothermal performance for isothermal chamber. Chin. J. Mech. Eng..

[20] Imamura K, Horinouchi O, Okuyama T, Isobe Y (2020). The primary dynamic gravimetric system for gas mass flow measurement. Flow Meas. Instrum..

[21] Kashan MAM, Leong A, Saha T, Kalavally V, Swamy V, Ramakrishnan N (2019). QCM-micropillar-based coupled resonators in the detection of gas mass flow rates. IEEE Trans. Instrum. Meas..

[22] GB/T 14513-1993. *Pneumatic Fluid Power-Determination of Flow Rate Characteristics of Pneumatic Component*. (1993)

[23] Hui W, Xu W, Jiang W (2020). Optimal selection of auxiliary components in serial connection method. Mach. Tool Hydraul..

[24] Zhang S, Xu W (2013). Study on critical pressure ratio and effective area. Hydraul. Pneum. Seals..

[25] Gao L, Yang G, Li W, Li B (2015). Measurement of mass flow rate and evaluation of heat transfer coefficient for high-pressure pneumatic components during charge and discharge processes. Flow Meas. Instrum..

[26] Li B, Gao L, Yang G (2013). Evaluation and compensation of steady gas flow force on the high-pressure electro-pneumatic servo valve direct-driven by voice coil motor. Energy Convers. Manag..

[27] Syah R, Elveny M, Nasution MKM (2021). Numerical investigation of nanofluid flow using CFD and fuzzy-based particle swarm optimization. Sci. Rep..

[28] Shoghl SN, Naderifar A, Farhadi F (2021). A novel strategy for comprehensive optimization of structural and operational parameters in a supersonic separator using computational fluid dynamics modeling. Sci. Rep..

[29] Molkov V, Dadashzadeh M, Makarov D (2019). Physical model of onboard hydrogen storage tank thermal behaviour during fueling. Int. J. Hydrog. Energy.

[30] Kawano Y, Kuroki T, Sakoda N, Monde M, Takata Y (2019). Thermal analysis of high-pressure hydrogen during the discharging process. Int. J. Hydrog. Energy.

[31] Bauer, T. *Thermophotovoltaics: Basic Principles and Critical Aspects of System Design*. 85–99. https://link.springer.com/book/10.1007%2F978-3-642-19965-3. (Springer, 2011).

[32] Gao L, Wu C, Zhang D, Fu X, Li B (2019). Research on a high-accuracy and high-pressure pneumatic servo valve with aerostatic bearing for precision control systems. Precis. Eng..

